# Associations of gender inequality with child malnutrition and mortality across 96 countries

**DOI:** 10.1017/gheg.2016.1

**Published:** 2016-03-23

**Authors:** A. A. Marphatia, T. J. Cole, C. Grijalva-Eternod, J. C. K. Wells

**Affiliations:** 1 Department of Geography, University of Cambridge, Cambridge, UK; 2 Population, Policy and Practice Programme, Institute of Child Health, University College London, London, UK; 3 Institute for Global Health, University College London, London, UK; 4 Childhood Nutrition Research Centre, University College London Institute of Child Health, London, UK

**Keywords:** Child malnutrition, child mortality, economic growth, gender inequality

## Abstract

National efforts to reduce low birth weight (LBW) and child malnutrition and mortality prioritise economic growth. However, this may be ineffective, while rising gross domestic product (GDP) also imposes health costs, such as obesity and non-communicable disease. There is a need to identify other potential routes for improving child health. We investigated associations of the Gender Inequality Index (GII), a national marker of women's disadvantages in reproductive health, empowerment and labour market participation, with the prevalence of LBW, child malnutrition (stunting and wasting) and mortality under 5 years in 96 countries, adjusting for national GDP. The GII displaced GDP as a predictor of LBW, explaining 36% of the variance. Independent of GDP, the GII explained 10% of the variance in wasting and stunting and 41% of the variance in child mortality. Simulations indicated that reducing GII could lead to major reductions in LBW, child malnutrition and mortality in low- and middle-income countries. Independent of national wealth, reducing women's disempowerment relative to men may reduce LBW and promote child nutritional status and survival. Longitudinal studies are now needed to evaluate the impact of efforts to reduce societal gender inequality.

## Introduction

Annually, one in seven neonates (20 million worldwide) have low birth weight (LBW; <2500 g) and 1 in 4 (165 million) are stunted (low height-for-age) [1]. This poor nutritional status is strongly associated with morbidity and mortality risk, both in the short- and long term [2, 3]. Stunted girls are likely to remain short in adulthood, thereby perpetuating the trans-generational cycle of nutritional disadvantage [4]. More broadly, poor growth in early life reduces human capital, including educational attainment and earning potential, and increases susceptibility to non-communicable disease [2, 4–11].

According to the widely used framework of the United Nations Children's Fund (UNICEF) child malnutrition is the outcome of a complex causal process [12]. ‘Immediate causes’ include inadequate dietary intake and high infection rates. ‘Underlying causes’ include insufficient access to food, inadequate health infrastructure, poor care and feeding practices. ‘Basic causes’ include the lack of financial and socio-economic resources available to households (e.g. education and employment) and inadequate political will. Although it is recognised that many of these factors act via constraints on the mother, the potential importance for these outcomes of women's *status* in wider society relative to men receives little attention from policy-makers.

Current efforts to reduce LBW, child malnutrition and mortality concentrate largely on mitigating the immediate causes, as listed in the top ten priorities outlined by the 2008 Copenhagen Consensus [13], rather than targeting the underlying or basic factors [14, 15]. Although some have recommended increasing economic and agricultural growth [16–20], analysis suggests that this approach is unlikely to be effective [21–24], arguably because it fails to address the structural factors underlying inequitable resource allocation within countries [25, 26].

Conceptually, maternal phenotype or ‘capital’ is the physiological niche to which each child is exposed during the start of life [27, 28]. Studies have consistently linked maternal undernutrition (short stature, low body mass index (BMI), anaemia) with LBW and stunting in the offspring [4, 29–31]. Increased maternal education has also been associated with improved child nutrition and lower mortality [3, 32]. Women's autonomy and household decision-making are particularly important for children's health in South Asian countries [33], and interventions targeting these factors through women's groups have improved child survival and health [34].

In 36 low/middle-income countries (LMICs), Smith and colleagues estimated that if women and men had equal status in the household, the prevalence of underweight children under 3 years would decrease by 13% (13.4 million) in South Asia and 3% (1.7 million) in Sub-Saharan Africa [35]. This pioneering work highlights the importance of gender inequality for child health, but is restricted to within-country analysis, and is limited to societies with high gender inequality and low levels of gross domestic product (GDP).

Whilst gender inequality can be assessed at the level of the household and community, it is also important to consider the way that society is organised, and this is best addressed at the level of the nation, which reflects national policies, legislation, budgetary allocations and so on. At the broader level of society, gender norms and practices shape the social institutions that structure daily life, and hence may promote gender inequality in nutrition, health and education. Through legislation and budgetary allocations, states define what constitutes acceptable or legitimate behaviour at all levels of social organisation. Independent of their wealth, states can thus create, reinforce, exacerbate or diminish social inequalities, and hence influence the relative status of the two genders.

However, there is little understanding of whether efforts to improve women's ability to participate on an equal footing with men in society might be a novel avenue for promoting child health. The potential importance of national policies promoting female education, incorporating women into the political system and labour markets, and targeting health problems that specifically afflict women through their physiological role in reproduction, merit particular attention because they potentially connect societal values of women with key parameters of child health. Across 116 countries, a national marker of female empowerment (ratio of female to male life expectancy at birth) was associated with reductions in stunting [21]. Similarly, Brinda *et al*. showed that the Gender Inequality Index (GII), which provides a national composite indicator of women's status in society relative to men, was associated with neonatal, infant and childhood mortality across 138 countries [36].

We therefore conducted a more comprehensive analysis of associations of societal gender inequality with variability across countries in child survival and malnutrition, taking into account GDP. This allows us to ask if two countries with similar national wealth, but that accord different status to women, have different levels of LBW, child malnutrition and survival. We also used our statistical models to simulate the potential effects of economic growth and reducing gender inequality on child survival and malnutrition.

## Methods

### Child-related outcomes

Country-specific data on mortality rate and stunting and wasting (low weight-for-height) prevalence for children under five years were compiled from the Human Development Report (HDR) 2011 (http://hdr.undp.org/en/reports/global/hdr2011/) [37]. Country-specific mortality rates were available for the year 2008, and stunting and wasting prevalences were available for the years 2000–2009.

Recent national estimates of LBW prevalence were obtained from UNICEF's database (http://www.childinfo.org/low_birthweight_table.php). As birthweight data are underreported in many countries, these prevalences should be treated with caution [1, 38, 39].

### Socioeconomic and gender inequality exposures

Data on GDP as an index of national wealth *per capita* for the year 2009 were compiled from the HDR 2011 [37]. To index women's status in society we used the recently developed GII, which had data for 100 countries in the HDR 2011, hence we searched for equivalent data on child health outcomes in these countries [37]. We also obtained GII data for 2008 and 2013, to ascertain the actual magnitude of change over this 5-year period. The GII is a new composite index (replacing and combining the previous Gender Empowerment Measure (GEM) and Gender Development Index (GDI)) measuring women's disadvantage in three dimensions: reproductive health, empowerment and the labour market [40]. The reproductive health dimension is based on two indices: the maternal mortality ratio and the adolescent fertility rate. The empowerment dimension is based on the share of parliamentary seats held by each sex, and gender differences in secondary and higher education attainment levels. The labour dimension reflects women's participation in the work force based on the International Labour Organization's Key Indicators of the Labour Market. GII values range from 0 (maximum equality) to 1 (maximum inequality).

The GII is intended to measure losses in human development due to societal gender inequality [40]. The GII score characterises where a country lies in reference to normative ideals for key indicators of women's reproductive health, and gender differences in empowerment and economic status.

### Anaylses and statistics

The primary exposures were the GII and GDP, and the primary outcomes were child mortality and the prevalences of LBW, stunting, wasting. We also investigated whether any of the nutritional status prevalence indicators, namely wasting, stunting and LBW, might mediate the association between exposures and outcomes, where these indices were not themselves the outcome, and where they might precede in time the outcome being investigated.

GDP, child mortality and the prevalences of LBW, stunting and wasting were all right-skewed, and were log10-transformed prior to analysis. GII was slightly left-skewed and was left untransformed. Preliminary analysis demonstrated significant correlations between GII and log GDP; however, the strength of this correlation was not sufficient to cause problems with collinearity, demonstrated by analysis of the variance inflation factor. We categorised countries according to geographical region, loosely based on the criteria of the World Health Organization, in order to assess regional patterns of GII and GDP using graphic analysis.

Associations between the variables were initially explored with Spearman rank correlation. The primary outcomes were then regressed in turn on GDP, with GII subsequently added. For stunting, wasting and child mortality, LBW was tested as a possible mediating factor. For child mortality, stunting and wasting were similarly tested. Many of the associations were highly significant, so *t*-statistics and partial correlations are presented with the regression models to indicate the strength of associations between variables and GII. Partial correlations were used to quantify the proportion of variance in the dependent variable explained by the GII adjusting for covariates such as GDP.

### Modelling

These regression models were then used to simulate the potential impact of changes in GDP and GII on LBW, stunting and mortality. We predicted outcome values for a country of a given GDP or GII centile value, and then simulated the effect of changes in GII or GDP values. We first simulated the effect of changes in GDP only, by using models that did not contain GII. We then used models incorporating both GDP and GII, in order to simulate the effects of: (a) economic growth in the absence of changes in gender inequality, (b) improvements in gender inequality in the absence of economic growth, and (c) improvements in both factors concurrently. For these simulations we calculated centiles for GDP and GII in our sample, using the relevant Excel Function (Microsoft Excel 2011 version 14.5.3). We simulated raising the GDP of low-income countries to middle-income country status (10th to 50th centile), and reducing the GII of low- or middle-income countries from the 90th to the 10th centile.

## Results

The characteristics of the sample are given in Table 1. We obtained matching data on GII and child health outcomes for 96 countries, except for LBW where there were two missing values, reducing the sample to 94 countries. Table 2 gives the correlations between the variables. GDP was negatively associated with all the other variables, including GII. GII was directly associated with the prevalences of LBW, stunting and wasting, and with child mortality rate. In 23 of the 96 countries, more than one in ten children dies before 5 years.

**Table 1.** Description of the data

Variable	*n*	Median	Interquartile range	Minimum	Maximum
Gender Inequality index (GII)	96	0.49	0.22	0.09	0.77
GDP (US$)	96	4330	7340	319	50 633
LBW (%)	94	11.0	6.0	3	34
Stunting (%)	96	27.5	24.0	1.3	63.1
Wasting (%)	96	10.3	16.0	0.5	43.5
Mortality under 5 years (per 1000)	96	37.5	78.0	3	209

**Table 2.** Spearman rank correlations between variables

	GII	GDP	LBW	Stunting	Wasting
GDP	−0.72				
LBW	0.64	−0.44			
Stunting	0.69	−0.79	0.48		
Wasting	0.69	−0.77	0.66	0.89	
Mortality	0.83	−0.84	0.54	0.78	0.74

GII, Gender inequality index; GDP, *Per capita* gross domestic product; LBW, low birth weight.

All variables except GII log-transformed.

All correlations significant *p* < 0.0001.

Box plots of GII and GDP by geographic region are given in online Supplementary Fig. S1. This shows that Sub-Saharan Africa and South Asia stand out as regions of high gender inequality, whereas East and West Sub-Saharan Africa and South Asia stand out as regions with the lowest levels of GDP.

### Association of GDP and GII with the outcomes

**Fig. 1.**
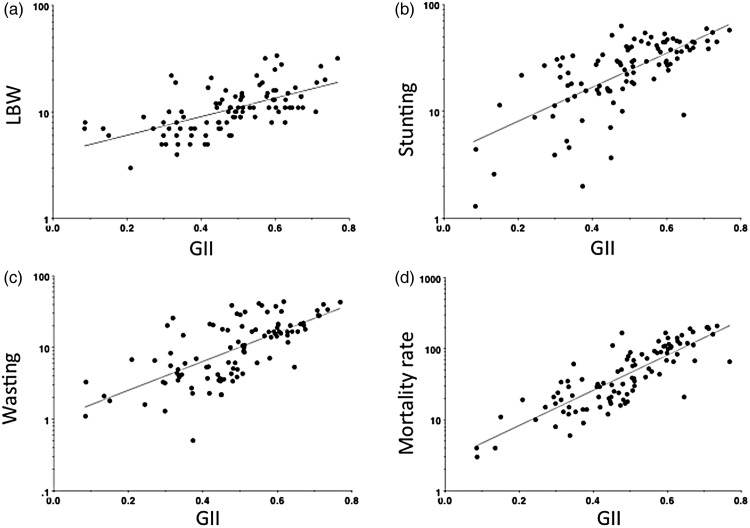
Associations of the GII and the prevalence of (*a*) LBW, (*b*) stunting, (*c*) wasting and (*d*) child mortality rate in 96 countries (two missing data points for LBW).

Results for the regression models of LBW, wasting and stunting prevalence on GDP and GII are given in Table 3. LBW was inversely associated with GDP (*t* = −4.0) but much more strongly and positively associated with GII (*t* = 7.3). The association between GII and LBW is given in Fig. 1*a*.

**Table 3.** Regression models of low birth weight, wasting and stunting prevalence, and mortality on GDP and GII

Outcome	Predictors	*B*	s.e.	*t*	*p*	*r*^2^	Partial *r*^2^
Low birth weight	GDP	−0.17	0.04	−4.0	0.0001	0.14	
	GII	0.87	0.12	7.3	<0.0001	0.36	
Stunting	GDP	−0.53	0.05	−11.2	<0.0001	0.57	
	GDP	−0.38	0.06	−5.9	<0.0001	0.61	0.27
	GII	0.67	0.21	3.2	0.002		0.10
Wasting	GDP	−0.70	0.06	−12.7	<0.0001	0.58	
	GDP	−0.50	0.08	−6.3	<0.0001	0.62	0.30
	GII	0.84	0.26	3.2	0.002		0.10
Mortality	GDP	−0.75	0.05	−15.4	0.0001	0.71	
	GDP	−0.45	0.05	−8.2	<0.0001	0.83	0.42
	GII	1.41	0.18	8.0	<0.0001		0.41
	GDP	−0.38	0.06	−6.0	<0.0001	0.84	0.28
	GII	1.29	0.18	7.0	<0.0001		0.35
	Stunting	0.18	0.09	2.1	0.034		0.05

GII, Gender inequality index; GDP, *Per capita* gross domestic product (US$); S.E., standard error.

All variables except GII log-transformed.

GDP was inversely associated with stunting both on its own (*t* = −11.2) and with GII, GDP (*t* = −5.9) being more predictive than GII (*t* = 3.2). GII explained 10% of the variance in stunting independent of GDP. LBW added to the model was not significant, indicating that it was not a strong mediator of the association between gender inequality and stunting. The association between the GII and stunting is given in Fig. 1*b*.

GDP was inversely associated with wasting both on its own (*t* = −12.7) and with GII, GDP (*t* = −6.3) being more predictive than GII (*t* = 3.2). GII explained 10% of the variance in wasting independent of GDP. Again LBW added to the model was not significant, and hence did not mediate the association between gender inequality and wasting. The association between GII and wasting is given in Fig. 1*c*.

GDP was inversely associated with mortality rate both on its own (*t* = −15.4) and with GII (Table 3), the two being similarly predictive (GDP: *t* = −8.2; GII: *t* = 8.0). GII explained 41% of the variance in mortality independent of GDP. Testing for potential mediating factors, stunting was also a significant predictor, whereas LBW and wasting were not. In this extended model GII explained 35% of the variance. The association between GII and child mortality rate is given in Fig. 1*d*.

### Modelling

Using the equation based only on GDP, raising GDP of low-income countries from the 10th to the 50th sample centile would reduce the prevalence of LBW from 13.2% to 10.4%. Alternatively, reducing gender inequality from the 90th to the 50th to the 10th centile would reduce the prevalence of LBW from 14.2% to 10.2% to 7.1%.

**Fig. 2.**
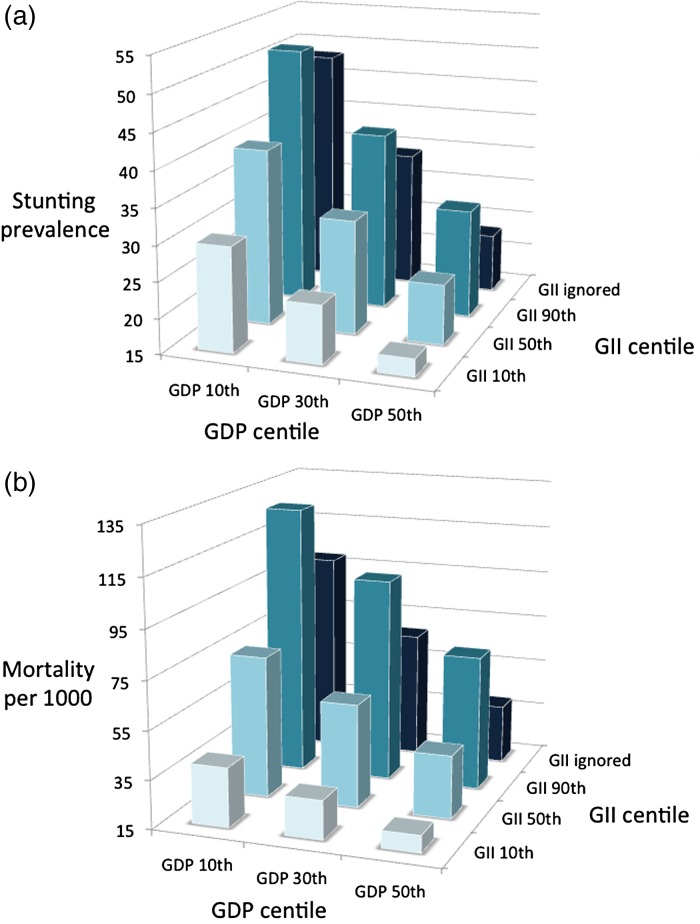
(*a*) Simulated changes in the prevalence of stunting expected from increasing GDP of a country from the 10th to the 50th centile, either in the absence of any change in GII, or in combination with reducing GII from 90th to 10th centile. (*b*) Simulated changes in child mortality rate expected from increasing GDP of a country from the 10th to the 50th centile, either in the absence of any change in GII, or in combination with reducing GII from 90th to 10th centile. Modelling based on regression equations in Table 3.

The results of the simulation for stunting are shown in Fig. 2*a*. Ignoring gender inequality, raising GDP of low-income countries from the 10th to the 50th centile would reduce stunting by half, from 48% to 23%. Taking gender inequality into account, raising GDP from the 10th to the 50th centile would have a smaller effect, reducing stunting from 51% to 30% for countries on the 90th centile for gender inequality, and from 39 to 23% for countries on the 50th centile for gender inequality. In low-income countries (10th centile GDP), reducing gender inequality from the 90th to the 50th to the 10th centile would decrease stunting from 51% to 39% to 30%. In middle-income countries (50th centile GDP), the same reduction in gender inequality would decrease stunting from 30% to 23% to 17%.

Ignoring gender inequality, raising GDP from the 10th to the 50th centile would reduce the prevalence of wasting by two thirds, from 24% to 9%. Taking gender inequality into account, raising GDP of low-income countries from the 10th to the 50th centile would have a smaller effect, reducing wasting from 26% to 13% for countries on the 90th centile for gender inequality, and from 19% to 9% for countries on the 50th centile for gender inequality. In low-income countries (10th centile GDP), reducing gender inequality from the 90th to the 50th to the 10th centile would decrease wasting by half, from 26% to 19% to 13%. In middle-income countries (50th centile GDP), the same reduction in gender inequality would decrease wasting from 13% to 9% to 6%.

The results of the simulation for child mortality are shown in Fig. 2*b*. Ignoring gender inequality, raising GDP of low-income countries from the 10th to the 50th centile would reduce mortality from 99 to 39 per 1000 (‰). Taking gender inequality into account, raising GDP from the 10th to the 50th centile would have a smaller effect, reducing mortality from 127‰ to 71‰ for countries on the 90th centile for gender inequality, and from 74‰ to 41‰ for countries on the 50th centile. In low-income countries (10th centile of GDP), reducing gender inequality from the 90th to the 50th to the 10th centile would reduce mortality from 127‰ to 74‰ to 40‰, while in middle-income countries (50th centile of GDP), the same reduction in gender inequality would reduce mortality from 71‰ to 41‰ to 22‰.

Between 2008 and 2013, median GII across 88 countries with data at both time points decreased from 0.53 to 0.48, equivalent to a shift from the 62nd to the 46th centile based on 2008 values.

## Discussion

Several studies have previously reported associations of child malnutrition with markers of women's status in the household or community [30, 33, 35]. These analyses have generally been restricted to LMICs with relatively high levels of gender inequality, as measured by the GII. The relative importance of societal gender norms and practices, reflecting the national organisation of healthcare, education, political participation and employment, has not been addressed.

We therefore bring a new perspective, by addressing a wider range of societies, and by focusing on indices of women's status that summarise the extent to which society (a) ameliorates the health risks imposed on women by physical reproduction, and (b) promotes gender parity in access to education, work opportunities, and participation in national policy-making. Our analysis of 96 countries shows that, independent of GDP, gender inequality at this broader societal level explains a substantial proportion of the variance in LBW, child malnutrition and mortality.

The potential importance of societal gender inequality is highlighted by our finding for LBW, where the GII explained 36% of the variance across countries and was more predictive than GDP. Independent of GDP, GII also explained 10% of the variance in wasting and stunting, and 41% of the variance in child mortality, and its inclusion in statistical models decreased the explanatory power of GDP. An association of the GII with neonatal, infant and childhood mortality was reported previously [36], but we now show that this association is relatively independent of LBW and child malnutrition, even though the latter are also strongly associated with the GII. While Brinda *et al*. linked the GII specifically with early mortality from infections such as diarrhoea and pneumonia [36], we show that gender inequality also impacts those who escape early mortality, through stunting and LBW. In this way, societal gender inequality may lead to long-term deficits in health and human capital [2].

Building on our regression models, our simulations suggest that reducing gender inequality would benefit child outcomes most strongly in the poorest countries. Shifting from the 90th to 50th GII centile in a poor country (10th centile of GDP) would decrease the prevalence of LBW by 4%, stunting by 10%, and childhood mortality by 54‰. To achieve similar gains by economic growth alone, these low-income countries would effectively need to become middle-income countries, shifting to the 50th centile of GDP.

Nevertheless, the potential of economic growth to improve child health remains controversial and poorly understood. First, whilst some longitudinal analyses have linked GDP growth to decreases in childhood stunting and maternal underweight [15, 16], others found little reduction in the prevalence of LBW and child malnutrition [14, 41–43]. Vollmer *et al*. suggest that economic growth is not as beneficial as previously believed, in part because the unequal distribution of growth in low-income countries means that wealth does not reach the poor and undernourished [22, 23].

Second, there is growing evidence that GDP represents a poor index of the social and economic factors that may impact child health [44]. GDP reflects the marketisation of services. It does not measure household-level income distribution, or living standards. When GDP rises, which tends to be primarily through greater male productivity, one cannot assume that the additional wealth is accessed in equal proportion by women, nor that it improves child health [45].

Third, GDP does not include unremunerated women's domestic activities that are especially relevant to child wellbeing [46–49]. Paradoxically, these same activities are closely associated with women's low status in society [50]. Lack of access to paid employment reduces women's control over household finances, and hinders their ability to direct resources to child welfare. This scenario may help explain why, independent of GDP, the GII explained so much of the variance in LBW and child mortality.

Fourth, any benefits of economic growth must be set against possible adverse health consequences. Among poorer countries, increasing GDP, market integration and foreign direct investment have all been associated with an increased prevalence of non-communicable disease [51]. These diseases are increasingly relevant to child health, as maternal obesity, diabetes and hypertension adversely affect foetal growth [52]. Notably, we have previously shown that the GII is positively associated with a ‘female’ excess in the prevalence of adult obesity [53]. Thus, unlike economic growth, gender parity may offer a unified approach for promoting nutritional health at all ages, reducing LBW, child malnutrition and adult non-communicable diseases.

The availability of comparable country-level data on GDP, GII and child health outcomes for a large sample of countries is strength of our analysis. However, there are also some limitations.

First, as with other ecological analyses cited, data are not available on intra-country or individual-level variability in these factors. The GII does not fully capture the constraints on women's decision-making power (at household and community levels), the ‘unpaid care work’ performed largely by women in the home or more subjective aspects of inequality, such as the pathways and processes that underlie it [54]. Thus, our associations between GII and children's mortality and malnutrition remain conservative.

Second, the GII has been available for only a few years. We are restricted to a cross-sectional analysis that cannot demonstrate causal associations, and might be prone to the ‘ecological fallacy’. We can only simulate potential longitudinal effects, though we have shown that GII values have on average declined over 5 years. Policies reducing gender inequality, such as promoting women's literacy or greater parliamentary representation, are expected to take time to affect gender inequality as experienced by individual women. These efforts require not only changes in national legislation and budgetary allocation, but also a shift in societal attitudes. Nevertheless, our findings, coupled with those of Brinda *et al*. [36], suggest that longitudinal evaluations merit undertaking.

Third, it is important to note that data on maternal mortality are incorporated in the GII. One component of maternal mortality is obstructed labour, and populations with high levels of obstructed labour may have undergone selection against higher birth weight [55]. However, maternal mortality is only a minority component of the GII, and LBW did not explain associations of GII with childhood malnutrition or mortality. This suggests that confounding by maternal mortality is not a major concern in our findings.

Fourth, the GII incorporates both absolute women-specific indicators (reproductive health) and relative (women *v.* men) indicators into a single formula, potentially creating methodological problems [56]. Nevertheless, indices such as the GII provide valuable data for quantifying the multiple dimensions of gender inequality for monitoring progress and identifying potential policy solutions [54].

In conclusion, our analysis and simulations suggest that efforts to promote women's ability to participate on an equal footing with men in society might have substantial benefits for children's health and survival, especially in LMICs. Crucially, such efforts may also reduce obesity and non-communicable diseases. The value of the GII is that it identifies specific capabilities and opportunities of women that interventions might target in order to accelerate progress in terms of their own wellbeing, children's health and human capital in general. High GII values reflect the widespread neglect of health, nutrition and other interests central to women, which not only harm women themselves, but also impose a burden on wider society [29].

## Supplementary material

For supplementary material accompanying this paper visit http://dx.doi.org/10.1017/gheg.2016.1. click here to view supplementary material
