# Supplementary material for: Associations of gender inequality with child malnutrition and mortality across 96 countries
Source: Glob Health Epidemiol Genom. 2016 Mar 23;1:e6. doi: 10.1017/gheg.2016.1 (PMC5870432; doi:10.1017/gheg.2016.1)
Supplement: Supplementary file 1 [file S2054420016000014sup001.docx]

**Supplementary Online Figure 1. Regional variability in GII (upper panel) and GDP (lower panel)**


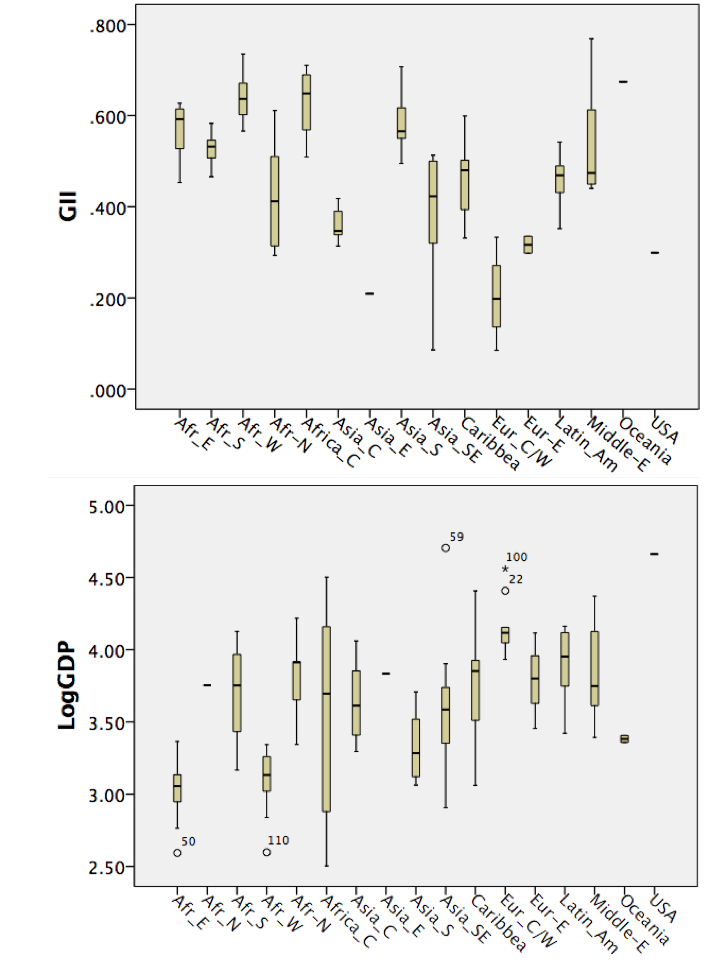


Regions loosely based on WHO criteria. Note that Central/Western Europe includes only one Western European country (Germany); China is the only East Asian country, and Singapore has been included in the South East Asia region.
